# Physiological synchrony is associated with cooperative success in real-life interactions

**DOI:** 10.1038/s41598-020-76539-8

**Published:** 2020-11-12

**Authors:** F. Behrens, J. A. Snijdewint, R. G. Moulder, E. Prochazkova, E. E. Sjak-Shie, S. M. Boker, M. E. Kret

**Affiliations:** 1 Cognitive Psychology Unit, Institute of Psychology, Leiden University, Wassenaarseweg 52, Leiden, 2333 AK The Netherlands; 2 Leiden Institute for Brain and Cognition (LIBC), Albinusdreef 2, Leiden, 2300 RC The Netherlands; 3 Department of Psychology, University of Virginia, Charlottesville, VA 22903 USA

**Keywords:** Social behaviour, Psychology

## Abstract

Cooperation is pivotal for society to flourish. To foster cooperation, humans express and read intentions via explicit signals and subtle reflections of arousal visible in the face. Evidence is accumulating that humans synchronize these nonverbal expressions and the physiological mechanisms underlying them, potentially influencing cooperation. The current study is designed to verify this putative linkage between synchrony and cooperation. To that end, 152 participants played the Prisoner’s Dilemma game in a dyadic interaction setting, sometimes facing each other and sometimes not. Results showed that synchrony in both heart rate and skin conductance level emerged during face-to-face contact. However, only synchrony in skin conductance levels predicted cooperative success of dyads. Crucially, this positive linkage was strengthened when participants could see each other. These findings show the strong relationship between our bodily responses and social behavior, and emphasize the importance of studying social processes *between* rather than within individuals in real-life interactions.

## Introduction

Cooperation is one of human society’s core pillars, distinguishing us from other species in its scale and complexity^1^. Despite countless examples of tremendous successes of people working together towards a common goal, there are as many examples where cooperation fails. An important question therefore is: How can cooperation be achieved? In order to be able to foster cooperation, we must first understand the mechanisms. The current study takes a step in that direction.

When making decisions, such as whether to cooperate or not, people rely on a variety of nonverbal expressions to communicate their own and predict others’ intentions^2,3^. Cooperation is risky as individuals can take advantage of those investing time and resources, and nonverbal expressions reflecting a person’s benign intents can help ensure cooperative success. Intriguingly, research has shown that emotional states tend to synchronize between interaction partners on several levels including the behavioral^4^, neural^5^, and physiological level^6,7^. This is in line with the idea that emotional states are multidimensional constructs and that activation of one of these levels simultaneously activates the other levels^8^. Although some of these emotion-induced changes cannot be observed by the naked eye directly, people may perceive them indirectly through visual cues such as pupil size or a blush on the cheeks, and align their bodily responses accordingly^9^. Whether or not physiological synchrony is associated with cooperative decisions is a key question that has thus far remained unanswered.

Raising awareness of synchronized emotion states has had a vast impact on different disciplines with researchers investigating its clinical^10^, developmental^11^, social^12^, evolutionary^13^, neural^14^, and cognitive^15^ implications. It has been proposed that the *function* of this alignment is to infer the other person’s emotions, to empathize, and to provide subsequent consolation, help, or other prosocial behavior^16^. Despite the clear predictions regarding the function of synchrony, psychological research has thus far only investigated the benefits of synchrony in artificial settings with either participants interacting with virtual characters on a computer screen^17^, or two people interacting in cooperative compared to competitive contexts^18^. Thus far, no studies have been conducted to specifically investigate the direct link between synchrony and cooperative decisions.

To what extent are synchrony and cooperative success linked? This pivotal question has never been directly addressed before. We aim to close this knowledge gap, focusing on physiological synchrony because it is implicit, hard to control or regulate, and is a crucial component of emotion processing^19,20^. In psychology, the most commonly studied physiological responses are skin conductance level, a purely sympathetic nervous system response, and heart rate, which reflects both sympathetic and parasympathetic nervous system activity^19,21^. Previous research has shown that before people make a decision which they indicate by, for instance, pressing a button in an experiment, that decision is already reflected in their physiology^22,23^. We here focus on these two physiological measures, investigating whether they synchronize between interaction partners and if so, whether that relates to their cooperative success.

**Figure 1:**
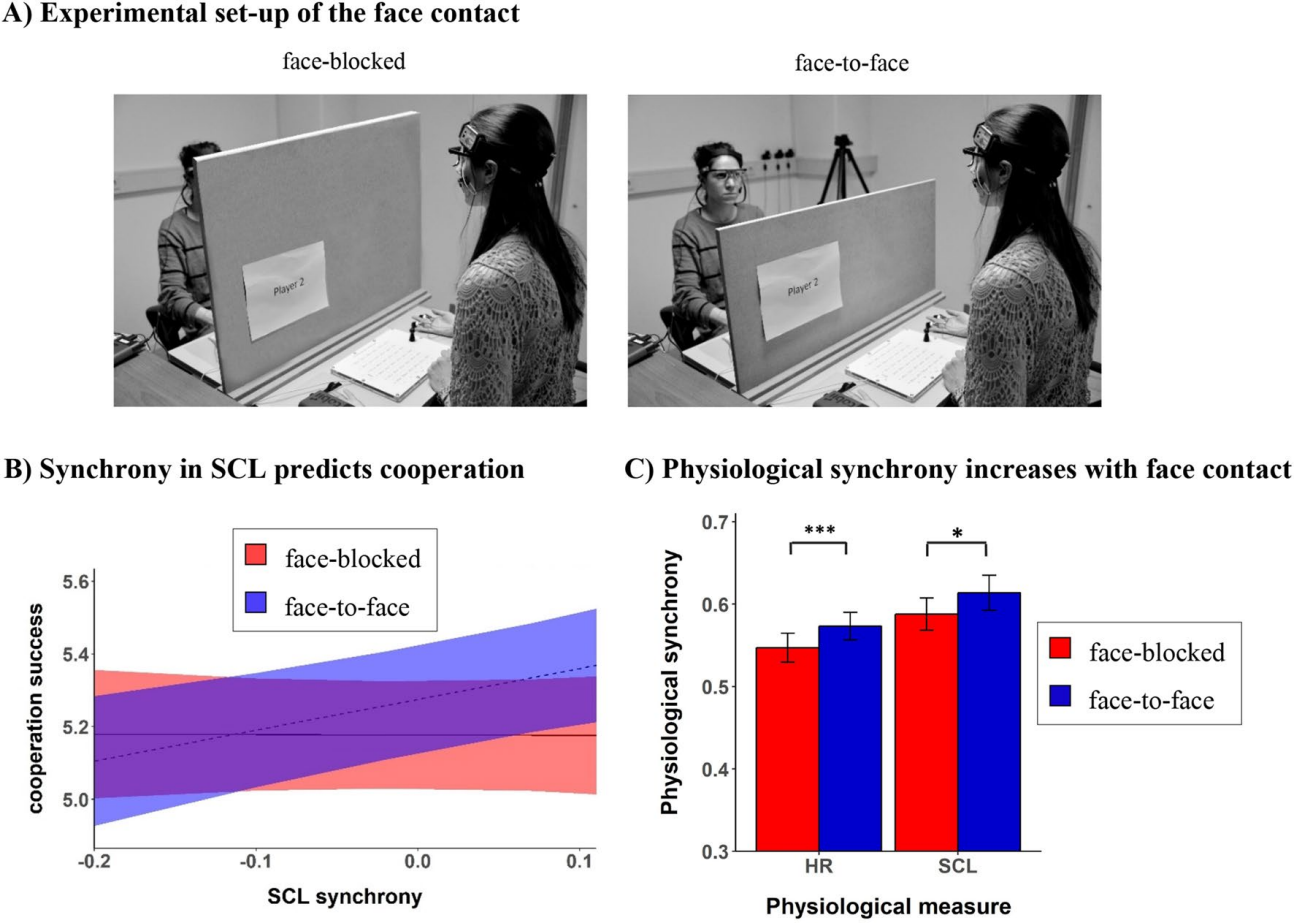
Experimental set-up and results. (**A**) Dyadic interaction in the face-blocked (left) and face-to-face (right) conditions. Inclusion of the two images was approved for publication by both individuals seen in the pictures and informed consent was obtained from both individuals to approve online open-access publication of these images. (**B**) Predicted values of cooperative success based on the interaction effect between synchrony in skin conductance level and Face condition. (**C**) Mean differences between the face-to-face (blue) and face-blocked condition (red) for heart rate and skin conductance level synchrony. The shaded areas in (**B**) and error bars in (**C**) represent 95%-confidence intervals. Physiological synchrony is measured by the mean windowed cross-correlation and is grand-mean centered for the analysis (see “Methods” section for details). Cooperative success is measured by the joint outcome of a dyad per trial in the economic game (range 4–6 points). *HR* heart rate, *SCL* skin conductance level. *p < 0.05; **p < 0.01; ***p < 0.001.

To that end, 152 naïve participants played a modified iterated Prisoner’s Dilemma game in dyads, 30 consecutive trials facing each other (face-to-face condition; allowing for nonverbal communication), and 30 consecutive trials with a visual cover between them, constraining them from interacting (face-blocked condition; the order of the two conditions was counterbalanced; see Panel A in Fig. 1 for a visualization of the two Face conditions). The original Prisoner’s Dilemma payoff structure where people can choose between two options (i.e., to cooperate or to defect) was extended to a 6 × 6 payoff structure to have a more fine-grained measure of cooperation (see “Methods” section). To quantify physiological synchrony, we conducted a windowed cross-correlation analysis which accounts for the non-stationarity of the time series and delays between individuals’ responses reflecting the dynamic nature of the interaction between two participants (see “Methods” section). The heart rate analysis included 60 dyads and the skin conductance level analysis 50 dyads (see “Methods” section)^24^. The aim of the study was twofold: First, we aimed to confirm that physiological synchrony emerges during dyadic interactions. Second, we aimed to investigate whether synchrony is related to cooperative success and whether such a relationship is bound to interactions where partners could see each other.

## Results

Investigating the joint outcome, the results showed that the interaction effect between skin conductance level synchrony and Face condition significantly predicted cooperative success (*t*(2882.33) = 3.24, *p* = 0.001, *f*^2^ = 0.013). As depicted in Panel B of Fig. 1, the interaction shows a positive slope in the case of face-to-face interactions (beta coefficient = 0.86) and a flat (very slightly negative) slope in the face-blocked condition (beta coefficient =  − 0.01). Thus, in line with our expectation, there was a positive relation between skin conductance level synchrony and cooperation when people could see each other, but not when they could not see each other. With regard to heart rate synchrony, results yielded no significant interaction effect with Face condition on cooperative success (*t*(2861.92) = 0.86, *p* = 0.389, *f*^2^ < 0.001). The VIF values were all smaller than 1.75, which is lower than the cut-off value of 5, suggesting that multicollinearity did not influence our results^25^. The full model summary is shown in Table S2 (see Supplementary Materials). In a post-hoc control analysis, we demonstrated that cooperative success could not be significantly explained by the two individuals’ independent arousal levels (*ps* > 0.10) suggesting that the effects of the current study cannot be explained by the mere arousal responses of the two individuals (see Supplementary Materials for more details and the model summary; Table S3). The VIF values were all smaller than 3.15 suggesting that multicollinearity did not influence our results^25^.

Other findings underscored the importance of face contact. Regarding the behavioral responses, participants were more successful in cooperating when they faced each other as compared to when they did not (*M*_*face*_ = 0.65; *M*_*face-blocked*_ = 0.59; *t*(3629.74) = 7.59, *p* < 0.001, *f*^2^ = 0.02; Fig. S2) (for similar findings, see^26,27^). With respect to physiological synchrony, as predicted, face-to-face contact amplified the level of synchrony in heart rate and skin conductance level (HR: *t*(59) = 3.76, *p* < 0.001, *f*^2^ = 0.24; SCL: *t*(49) = 2.40, *p* = 0.020, *f*^2^ = 0.12). See Panel C of Fig. 1 for the corresponding plots. Finally, in a control analysis, we compared the level of synchrony from the original dyads with newly generated, randomly matched dyads. Specifically, participants were paired with another partner than the one they had actually interacted with in the experiment. This analysis verified that the level of synchrony was due to the interaction rather than possible confounding factors such as the experimental set-up of the study. For both heart rate and skin conductance level, the original dyads showed significantly higher Fisher-Z transformed correlations than the newly generated dyads (HR: *t*(3622.7) = 8.06, *p* < 0.001, *d* = 0.27; SCL: *t*(3015.5) = 4.38, *p* < 0.001, *d* = 0.15). In the Supplementary Materials, we provide a more detailed description of the control analysis.

## Discussion

For thousands of generations, humans have cooperated with others on unprecedented scales, which has been essential for their survival^1^. However, as is clear when opening the newspaper, cooperation also often fails. The core question is: what is the mechanism underlying successful cooperation? The current study gives insight into this question by investigating whether cooperative success is related to interaction partners detecting nonverbal signals reflective of physiological arousal, emotionally converging, and fostering mutual understanding and trust. Specifically, the aim of the current study was to investigate the linkage between physiological synchrony and cooperation. For the first time in the literature, we demonstrate that physiological synchrony is associated with cooperative success in real-life interactions. Importantly, this link is especially pronounced when people face each other, that is, when people are able to exchange nonverbal signals. Interestingly, these effects are only evident for skin conductance level synchrony, but not heart rate synchrony. Furthermore, both physiological synchrony and cooperative success are higher when people face each other, and synchrony levels are higher in real compared to artificially-generated dyads. These findings imply that people can detect subtle changes in another person’s face, and react to these changes, which is positively associated with cooperation success. Physiological synchrony therefore acts as an unconscious mechanism that affects our behavior and improves the success of close social interactions.

Synchronization is observed on many different levels^9^, in infants^6,28^, and in different species^13,29,30^. Theoretically, it has been proposed to make two interaction partners more similar, aligned, and easier to predict, which is why they are able to cooperate more efficiently^16^. By manipulating a cooperative versus competitive context, previous research showed increased heart rate synchrony^31^ and skin conductance synchrony^32^ in a cooperative compared to a competitive context. The current study builds on this work by showing that when people could decide themselves on a trial-by-trial basis whether they wanted to cooperate or not, these decisions were positively associated with the level of synchrony. This new approach better reflects natural situations where multiple small decisions are taken and thus shows the true relationship between synchronization and cooperative success.

Cooperation carries the risk of exploitation by non-cooperators, therefore being able to detect the integrity of another person’s intent is crucial. These intentions are reflected in a variety of behavioral and physiological signals that are visible in the face^8^. This is supported by the current finding that people were more successful when they played face-to-face compared to when they could not exchange nonverbal signals. We observed a similar effect in a previous, separate study where we used the same set-up, but a new sample of participants played the classical instead of the extended Prisoner’s Dilemma game^27^. Here, we would like to note that in that study we also manipulated whether participants received feedback about each other’s decisions or not and, contrarily to the current study, observed a positive effect of feedback. Two methodological differences might have contributed to such discrepancy: (i) the payoff structure was extended from a 2 × 2 to a 6 × 6 response matrix, and (ii) while in the previous study, participants first made a decision about whether they wanted to cooperate or not and subsequently indicated what they thought the other person chose; in the current study, the decision and prediction were combined into one response (i.e., participants place a pawn in the payoff matrix where the x-axis represents their own decision and the y-axis indicates their prediction about the other participant’s decision). As these two factors are the most prominent changes to our previous study, we believe that they are likely candidates to explain the differences in findings. Coming back to the effect of face contact, people have been shown to be more willing to cooperate when they could talk face-to-face rather than write emails, again supporting the beneficial effect of nonverbal signals^26^. Although the positive effect of face-to-face contact on cooperation is well documented, is it less clear what it is exactly that elicits such effect.

Behavioral signals such as facial expressions and eye gaze can provide valuable information about the intentions of others. However, these signals can in principle be consciously controlled and therefore faked and do not necessarily reflect a person’s true intentions^2,33^. Physiological responses, on the other hand, are difficult to control and are indicative of social decision-making^3,19^. Synchronizing on the physiological level has been proposed to change the way Person A feels about and behaves towards Person B which is consequently reflected in signals visible to Person A^33^. Likewise, if the explicit signals do show benign intentions, such signals and their mimicry can influence autonomic responses and their synchrony implying a bi-directional interaction between autonomic cues and explicit signals. The influence of visible signals on the synchrony in heart rate and skin conductance level is supported by the current finding that people synchronized more when they interacted face-to-face compared to no face contact; visible signals could be exchanged in the former but not in the latter condition. Thus, we argue that cooperation flourishes when people synchronize their autonomic responses because they align emotional states based on genuine emotional cues that are perceived by interaction partners.

The question remains which emotional cues the observer perceives to pick up the changes in heart rate and skin conductance level which can lead to interpersonal synchrony in these measures. Besides pronounced signals such as facial expressions and eye gaze, other subtle, yet visible cues that are closely linked to changes in arousal are pupil dilation and blushing. It has been demonstrated that people can observe changes in blushing in another person’s face and that blushing increases trust, a precursor of cooperation^34,35^. In addition, changes in pupil size have been specifically linked to changes in skin conductance level, but not in heart rate^36^. Again, people have been observed to be sensitive to these pupil size changes in another person^37^ and to show more trust towards people with dilated pupils^15^. These studies suggest that visible physiological responses such as pupil dilation and blushing might constitute suitable candidates for emotional cues that people use to perceive and synchronize changes in arousal as reflected in heart rate and skin conductance level. However, future research is needed to draw strong conclusions about the underlying mechanisms of how physiological synchrony emerges.

Interestingly, we observed that only synchrony in skin conductance level, but not in heart rate affected cooperative success. Such specificity to the purely sympathetic response was not anticipated, but can potentially be explained from hindsight. Sympathetic synchrony has been shown to elicit perceived similarity between interaction partners^38^ and perceived similarity has been shown to foster cooperation^39^. Furthermore, the sympathetic changes in skin conductance level have been related to (disadvantageous) decision-making and emotion regulation^22,40^. Given the risk of being exploited during cooperation, one might need increased emotion regulation to control the urge to defect in order to successfully cooperate. “Clicking” with another person on the autonomic level might therefore be an essential component of cooperation. These suggestions are, however, speculative and future research is needed to draw strong conclusions about how different responses and their synchrony are integrated in affecting social decision-making.

Two crucial control analyses underscore that synchrony was more than the sum of the arousal responses of two individuals or an artifact of sharing the same environment (e.g., participating in the experiment, receiving the same instructions, etc.). First, it might be argued that if both participants cooperate, their skin conductance level will increase as a reflection of their own decision without any influence of the interaction partner. However, the fact that cooperative success could not be predicted based on participants skin conductance levels alone argues against such interpretation. Second, it might be argued that the increased synchrony levels observed in our study could be the result of a shared environment. However, this argument is confuted by the finding that synchrony was higher for people interacting with each other compared to dyads who shared the same environment, but never actually interacted. This strengthens the notion that synchrony elevated during the actual interaction rather than constitutes an artifact of being in the similar situation. Here, we would like to note that with ‘the similar situation’ we refer to the broader situation such as participating in the same experiment and hearing the same instructions. What is not captured by the two control analyses is the influence of sharing the same specific experience of, for example, making the same decision at the same time. Such shared experience is by definition created when cooperation succeeds, as both individuals need to decide to cooperate. However, the same is true for situations where both participants decide to defect. An important question is therefore whether the link between cooperation and synchrony goes beyond the shared experience of choosing the same response option. In that case, we would expect higher levels of synchrony when both people cooperate compared to when they both defect. We tried to run an additional control analysis to test this hypothesis, however, due to the fact that the data incorporate twice the number of mutual cooperation trials compared to mutual defection, we were not able to perform a valid analysis. Future research is therefore needed to investigate the effect of sharing the same experience on the observed association between synchrony and cooperation. Besides this open question, based on the two control analyses that we did perform, we are confident that the measure of physiological synchrony is the result of a social interaction and that interpersonal rather than intrapersonal processes drive the link with cooperation in the current study.

At this point we would like to clarify that we do not make any claims about the direction of the observed effects. Although some models, such as the Perception Action Model^16^, suggest that synchrony drives social perception, it could also be a reflection of social processes. The design of the current study, that is, people first look at each other before making the decision, is in line with the idea that synchrony drives cooperation. However, previous studies showing that manipulating a cooperative versus competitive context increased synchrony supports the opposite direction. Future studies should scrutinize the causal relation between synchrony and cooperation by manipulating both variables.

The current study has significant implications for studying the intricate dynamics of cooperation. We provide unique evidence that physiological synchrony plays a crucial role in how successful people cooperate. Studying cooperation in real-life interactions unfolded a new layer of communicative processes that is ignored when using computerized, one-person paradigms. This new layer incorporates how two bodies communicate on a subtle level that we are not aware of, yet that is related to how we behave towards other individuals. Shedding light onto what makes cooperation successful in healthy interactions can help us understand situations where human interactions fail. Conflict resolution, whether in a conversation, a company or an international collaboration, is dependent on moment-by-moment cooperative tendencies of its individuals. Such tendencies are by virtue reliant on human’s ability to understand each other’s emotions and on the capacity to balance their emotions with one another. Applying this to clinical populations, it has been suggested that the lack of interpersonal exchange of nonverbal signals underlies deficits evident in autism, social anxiety, and depression, insights that can advance new therapies in these populations^10,41^. Our findings broaden our understanding of the role of synchrony in social behavior and add a hereto forth missing piece to the puzzle of understanding the link between nonverbal communication and cooperation.

## Methods

### Participants

In total, 152 individuals participated in the study (71% females, *M*_*age*_ = 23, *SD*_*age*_ = 4.3), who were recruited via the University online recruitment system (SONA) and by approaching people on University ground. By the time of data collection, we were not aware of methods to calculate a priori power analyses for hierarchical data structures. Instead, we based the sample size on our previous studies, where we used a very similar set-up^27^. Although recent advances would make it possible to conduct a post-hoc power analysis, we refrain from this as it has been suggested to greatly depend on the p-value of the observed effects (for a detailed explanation, see e.g.,^42,43^). Instead, we conducted a sensitivity analysis which has been recommended as a valid post-hoc method^44^. In contrast to an a priori power analysis where the necessary sample size is calculated for a given power and effect size, the sensitivity analysis consists of simulation-based power analyses for different effect sizes with the fixed sample size of the study assuming that the effect sizes are the true population parameters. The results show that the minimum true effect that we can detect with a power of 80% and the sample size of our study (*N* = 50) is 0.70. The observed effect size of 0.86 is associated with a power of 89%, again assuming that the observed effect size reflects the true population effect size. Details on the sensitivity analysis and the associated power curve are described in the Supplementary Materials.

Participants were organized in dyads. A dyad consisted of two same-sex individuals who did not know each other *(N*_*dyads*_ = 76). The reason for including same-sex dyads only were that (i) factors such as sexual attraction could have influenced the level of synchrony in mixed-sex dyads^33^ and (ii) people have been shown to behave differently in social dilemma games when playing with their own compared to the other gender^45^. All participants had normal or corrected-to-normal vision wearing contact lenses (glasses were not compatible with the eye-tracking glasses, see below). They received either course credits or a monetary reward (8€) for participation and could earn an additional maximum of 2€ depending on their performance during the experiment (no deception). Informed consent was obtained from all participants (all participants were 18 years old or older). The study was approved by the Psychology Research Ethics Committee of Leiden University (CEP17-0113/18) and follows the relevant guidelines and regulations to conduct a study with human participants.

#### Missing data

For the behavioral data, three of the 152 participants (= 76 dyads) were excluded because they had missing data for 30 or more out of 60 trials. For the physiological data, the decision to exclude data was based on the manual preprocessing of the data. Either the measurement of the physiological responses was erroneous in at least one of the two participants during the whole session or more than 70% of the responses were missing due to local measurement errors in the data. Based on these criteria, 14 dyads had to be excluded. The reason for such high rates of measurement errors is that we measured multiple physiological responses wirelessly and the recording devices would sometimes lose the signal during the experiment. In addition, the synchrony level was computed on the dyadic level, therefore we needed to exclude both participants if one of them had inaccurate measurements. Two additional dyads were excluded because they did not make any eye-contact during the face-to-face condition trials which was verified by means of eye-tracking glasses worn during the experiment. Ten additional dyads were excluded from the skin conductance level analysis due to measurement errors. Thus, the heart rate analysis included 60 dyads and the skin conductance level analysis 50 dyads which lies in the upper range of sample sizes across studies investigating physiological synchrony^24^. In addition, 29 single trials for the heart rate data and three single trials for the skin conductance level data were excluded.

### Design

The objective of the study was to investigate whether cooperative success could be predicted based on the physiological synchrony between two individuals in a real-life interaction setting. To this end, two participants played a modified iterated Prisoner’s Dilemma game while their heart rate and skin conductance level were measured. A mixed-design study was conducted with one within-dyad (Face manipulation) and one between-dyad (Feedback manipulation) variable. In the latter manipulation, people received auditory feedback about their decision or not. However, this manipulation did not influence cooperation (*χ*^2^(1) = 1.29, *p* = 0.256), and was not the focus of this article. As such, the Feedback manipulation is not discussed and only included as a control variable in the analysis. Regarding the Face manipulation, participants could either see each other’s face (face-to-face condition) or they could not see each other (face-blocked condition). All dyads played a block of 30 rounds of the game in each condition with the order counterbalanced. The dependent variable was cooperation which was measured by means of a modified version of the Prisoner’s Dilemma game (see below). During the experiment, participants’ heart rate, skin conductance level and eye movements were measured.

### Materials

#### Cooperation game

To measure cooperation, a modified version of the Prisoner’s Dilemma game was used. The general idea of the game is that people can choose between two options (cooperate versus defect) that affect both a person’s own and the partner’s outcome. In particular, if both players cooperate (CC), each player receives more points compared to if both players defect (DD). If one player cooperates and the other defects, the latter receives the highest points possible, while the former receives the lowest points. Hence, the dilemma is to choose between maximizing the own outcome by defecting (which is more advantageous independent of the other player’s choice) or maximizing the joint outcome by cooperating (the highest joint outcome is achieved when both players cooperate). In the current study, the idea of the game stayed the same, but people could choose between six instead of two options (option A–F) creating a cooperation scale (Table 1). For this purpose, we built two boards where participants could put a pawn on the response matrix to indicate their response. That response incorporated two choices: (1) the level of willingness to cooperate; moving from the left (option A) to the right (option F) on the x-axis, the willingness to cooperate increased with option A reflecting full defection and option F reflecting full cooperation; (2) what the participant thought the other person would choose on that trial; moving from the bottom (option A) to the top (option F) on the y-axis indicates that the participant expected the partner to cooperate more. Hence, the highlighted options in the four corners in Table 1 reflect the payoff structure of a traditional Prisoner’s Dilemma game, but the extended matrix shows the innovative structure designed for the current experiment. We recently observed that behavior displayed in this extended version of the Prisoner’s Dilemma game positively correlated with the behavior shown in the classical Prisoner’s Dilemma game suggesting that they measure similar behavioral tendencies^46^.

**Table 1:** Payoff structure of the current study (bold numbers were not highlighted during the experiment).

Other	F	**4.0**–**1.0**	3.8–1.4	3.6–1.8	3.4–2.2	3.2–2.6	**3.0**–**3.0**
	E	3.6–1.2	3.4–1.6	3.2–2.0	3.0–2.4	2.8–2.8	2.6–3.2
	D	3.2–1.4	3.0–1.8	2.8–2.2	2.6–2.6	2.4–3.0	2.2–3.4
	C	2.8–1.6	2.6–2.0	2.4–2.4	2.2–2.8	2.0–3.2	1.8–3.6
	B	2.4–1.8	2.2 -2.2	2.0–2.6	1.8–3.0	1.6–3.4	1.4–3.8
	A	**2.0**–**2.0**	1.8–2.4	1.6–2.8	1.4–3.2	1.2–3.6	**1.0**–**4.0**
		A	B	C	D	E	F
	You						

The first number refers to the points earned by “You”.

#### Physiological data acquisition and preparation

Throughout the experiment, four physiological responses were measured on both participants: heart rate (HR), skin conductance level (SCL), zygomaticus major (smiling muscle) and eye movements by means of electrocardiography (ECG), electrodermal activity (EDA), electromyography (EMG), and eye tracking glasses, respectively. The former three were recorded wirelessly with the MP150 BIOPAC data acquisition system and sampled at 2000 Hz. The EMG data contained many artifacts where the source could not be identified and the shape of the artifacts did not allow for clear distinction between artifacts and responses. Therefore, the facial expression data were not included in this paper.

For the analyses, the preprocessed heart rate and skin conductance level measures were down-sampled to 20 Hz. The software AcqKnowledge (AcqKnowledge v. 4.4; BIOPAC Systems Inc.) was used to record and sync the signals from the physiological signals, the event markers from E-Prime which was used to present the instructions and lock the behavioral responses, and markers sent by the eye tracking glasses.

#### Heart rate

To measure participants’ heart rate, electrodes were attached on the left and right side of the abdomen and on the thorax below the right collar bone. To process the data, an in-house developed software, PhysioData Toolbox^47^, was used offline. The signals were band-filtered with a cut-off of 1 Hz and 50 Hz. The R-peaks that were automatically detected by the software were afterwards visually inspected and manually corrected in case of missed or incorrect R-peaks. To still generate a smooth and continuous heart rate signal, interbeat intervals (IBI) were linearly interpolated in these locations. Participants with less than 30% coverage of the sum of the IBIs relative to the duration of the time signal were excluded. The signal used for the analyses was heart rate which was measured in beats-per-minutes.

#### Skin conductance level

Two electrodes were attached on the intermediate phalanges of the index and ring finger of the non-dominant hand. To improve the quality of the signal, there was a time interval of around 15 min between the attachment of the electrodes and the beginning of the data collection. The skin conductance level measures were low-pass filtered with a cut-off of 5 Hz and subsequently visually inspected for artifacts using the PhysioData Toolbox^47^.

#### Eye movements

Participants were wearing Tobii Pro Glasses 2 to track their eye movement and to verify whether they were looking at each other during the face-to-face condition trials. Fixation points were manually coded in Tobii Lab Pro (version 1.64, 2017). Trials in which participants were not at least once looking at the face of the other person were excluded.

We would like to note that the initial plan was to analyze the eye fixation data and the pupil data that were also measured with the Tobii Pro Glasses 2 in the current manuscript. Unfortunately, the data was of insufficient quality. With regard to the eye fixation data, the reason for the low quality is that in order to know where people looked at, we manually coded people’s eye fixations (i.e., did they look at the partner’s eyes, nose, mouth, shoulder, or at the background). The location of the fixation was indicated by a circle in the videos of the eye-tracking glasses. However, the circle was rather large in relation to the size of the face of the other person because participants sat on a table with some distance between them. The resolution of where participants looked exactly was therefore not ideal. For that reason, we decided to only use the data to exclude trials where participants did not look at the other person at all. Here, we used a rather liberal exclusion criteria where trials were only excluded if participants did not look once at the other person. With regard to the pupil size data, participants continuously changed their head position as they were instructed to look up to see their partner and to look down to make the decision. As a consequence, pupil size data were mostly missing during the decision time and were strongly influenced by luminance changes during the four seconds that people looked at each other. Additionally, there was a difference in luminance between the face-to-face condition and the face-blocked condition.

### Procedure

Before participants came to the lab, they received information about the study and filled out three questionnaire about empathy (Interpersonal Relation Index; IRI)^48^, social anxiety (Liebowitz Social Anxiety Scale; LSAS)^49^, and social value orientation (SVO)^50^. Upon arrival at the lab, participants signed an informed consent in separate rooms and a female researcher attached the electrodes for measuring heart rate, skin conductance level, and facial expressions (see “Methods” section). Next, participants filled out the Positive and Negative Affect Scale (PANAS)^51^ and read the instructions for the social dilemma game. Their understanding of the game was checked with multiple choice questions which were discussed in more detail when answered incorrectly. Afterwards, both participants sat on a table in front of each other with a wooden board between them such that they could only see each other’s faces. Finally, the eye tracking glasses were calibrated, the researcher left the room and started the experiment.

After three practice trials (always in the face-to-face condition), participants played the game two times, 30 rounds in the face-to-face and face-blocked condition. The order of starting in one or the other condition was counterbalanced. To block nonverbal communication in the latter condition, a visual cover was placed on top of the wooden board. The sequence of the trial was as follows with auditory instructions given via speakers: First, participants were instructed to look at each other (look at the cross in front of them [drawn on the visual cover] in the face-blocked condition). After 4 s, they were asked to look down and make a decision. When both individuals made their decision, they either heard that they both made a decision (no feedback condition) or heard how many points each player received based on their decisions (feedback condition). As mentioned above, the role of feedback is not discussed here and only added as a control variable in the analyses.

After each session, participants filled out a visual analogue scale (VAS) about their current feelings and experiences. After the second session, participants were separated again in different rooms where they filled out the Desire for Future Interaction scale (DFI)^52^ and read the debriefing form. Finally, they were paid and thanked for participation.

### Statistical analysis

During the study, different questionnaires about the participants’ characteristics and current mood were measured as mentioned in the Procedure. These data were not the focus of the current article and are not discussed any further. In the Supplementary Materials, we provide descriptive statistics of these questionnaires (see Table S1).

#### Behavioral data

We hypothesized that face contact would increase the joint outcome, i.e. cooperative success. Specifically, cooperative success was measured as the points both players earned together which ranged from 4.0 to 6.0 points. The Face condition variable was coded 0 = face-blocked condition and 1 = face-to-face condition. We conducted a multilevel linear regression analysis with dyads added as a random intercept effect. The inclusion of the random effect was verified by running an empty model consisting of the random effect only and calculating the intra-class correlation which quantifies how much dependency there is in the data. The significance level of 0.05 was applied. We report the *f*^2^ as a measure of effect size which is classified as small at a value of 0.02, medium at a value of 0.15, and large at a value of 0.35^53,54^. Dyads with more than 50% missing data (more than 30 trials) were excluded.

#### Physiological data

We conducted a lagged windowed cross-correlation analysis to quantify physiological synchrony for the heart rate and skin conductance level measures separately^55^. The objective of this analysis is to calculate the strength of association between two time series while taking into account the non-stationarity of the signals and the lag between responses, that is, to consider the dynamics of a dyadic interaction. Non-stationarity is accounted for by breaking down the time series into smaller windows (in the current study, the size of the windows is 8 s) and calculating the cross-correlation of each segment, allowing the correlation to change throughout the time series. These overlapping window segments are moved along the time series in steps of two seconds starting from the beginning to the end of each Face condition (i.e., moving along the 30 trials per condition). In addition, for each window segment, the signals of the two participants are lagged in relation to one another (in the current study, up to a maximum of four seconds in steps of 100 ms) allowing for differences in how fast people react to events and to one another^55^. For each window segment, the maximum cross-correlation (called ‘peak cross-correlation’) is detected across the different lags and subsequently, these maximum cross-correlations are averaged over all window segments within each Face condition. We therefore obtained a measure of the strength of synchrony for each Face condition per dyad. A more detailed description of the analysis can be found in the Supplementary Material (“Quantification of physiological synchrony”).

#### Hypothesis testing

Based on the synchrony measures we conducted two analyses to (i) investigate whether synchrony is influenced by the face contact manipulations, and (ii) test whether the joint outcome can be predicted based on synchrony and on whether people could see each other or not. For both analyses, multilevel linear regression analyses were performed with the same procedure as for the behavioral data. Regarding the first part, Face condition was added as the predictor and the synchrony measure for heart rate and skin conductance level responses as the outcome variables. For the second part, we ran one model with cooperative success as the outcome variable and the main effects and two-way interaction effects of the synchrony measures and Face condition as the predictors. Additionally, we included Feedback (feedback = 1; no feedback = 0) as a control variable. To check that multicollinearity does not confound our results, we calculated the variance inflation factor^25^.

## Supplementary information

Supplementary Information.

## Data Availability

All data, code, and materials that are associated with this paper and used to conduct the analyses will be uploaded and made accessible on the Leiden University archiving platform DataverseNL when published.
