# Supplementary material for: Physiological synchrony is associated with cooperative success in real-life interactions
Source: Sci Rep. 2020 Nov 12;10:19609. doi: 10.1038/s41598-020-76539-8 (PMC7661712; doi:10.1038/s41598-020-76539-8)
Supplement: Supplementary file 1 — Supplementary Information. [file 41598_2020_76539_MOESM1_ESM.docx]

Supplementary Materials for

Physiological synchrony is associated with cooperative success

in real-life interactions

F. Behrens, J. A. Snijdewint, R. G. Moulder, E. Prochazkova, E. E. Sjak-Shie,

S. M. Boker, M. E. Kret.

Corresponding author: M.E. Kret

Email: [m.e.kret@fsw.leidenuniv.nl](mailto:m.e.kret@fsw.leidenuniv.nl)

Sensitivity Analysis

The sensitivity analysis has been proposed to be a valid post-hoc analysis in case an a priori power analysis has not been conducted before the study ^54^. In contrast to the traditional power analysis, where the relationship between power and sample size given a specified effect size is computed, the sensitivity analysis investigates the relationship between power and effect size given a particular sample size. The idea is to run simulation-based power analyses and detect the minimum true effect size that a study is sensitive enough to detect given a certain level of power (mostly, 80%) and a specific sample size.

The simulation-based sensitivity analysis includes the following steps that are repeated 1000 times: (i) simulate new data for the response variables based on the specified model (in our case, the full model shown in Table S2); (ii) refit the model to the new data; (iii) perform a statistical test on the effect of interest (in our case, the interaction effect between skin conductance level synchrony and Face condition). The assumption is that the effect of interest reflects the true population effect size, so every positive test is a true positive and every negative test is a false negative (i.e., a Type II error). Based on these results, the power can be directly calculated from the number of successes and failures ^42^. This power analysis is not only performed for the observed effect (in our case, the estimated interaction effect between skin conductance level synchrony and Face condition [.86], see Table S2), but also for a range of other effect sizes. Notice that the effect size is based on the scaled estimate of the model rather than a standardized effect size. For each effect size, the power to detect that effect (assuming that it is the true population effect size) is calculated resulting in the curve shown in Figure S1. The dashed line indicates the 80% power criterion and its associated true effect size (.70) that we can detect given our sample size. In other words, with our design, we would find a significant p-value in 80% of the cases if the true effect size was .70. The observed effect size of .86 is associated with a power of 89 %, again assuming that the observed effect size reflects the true population effect size.


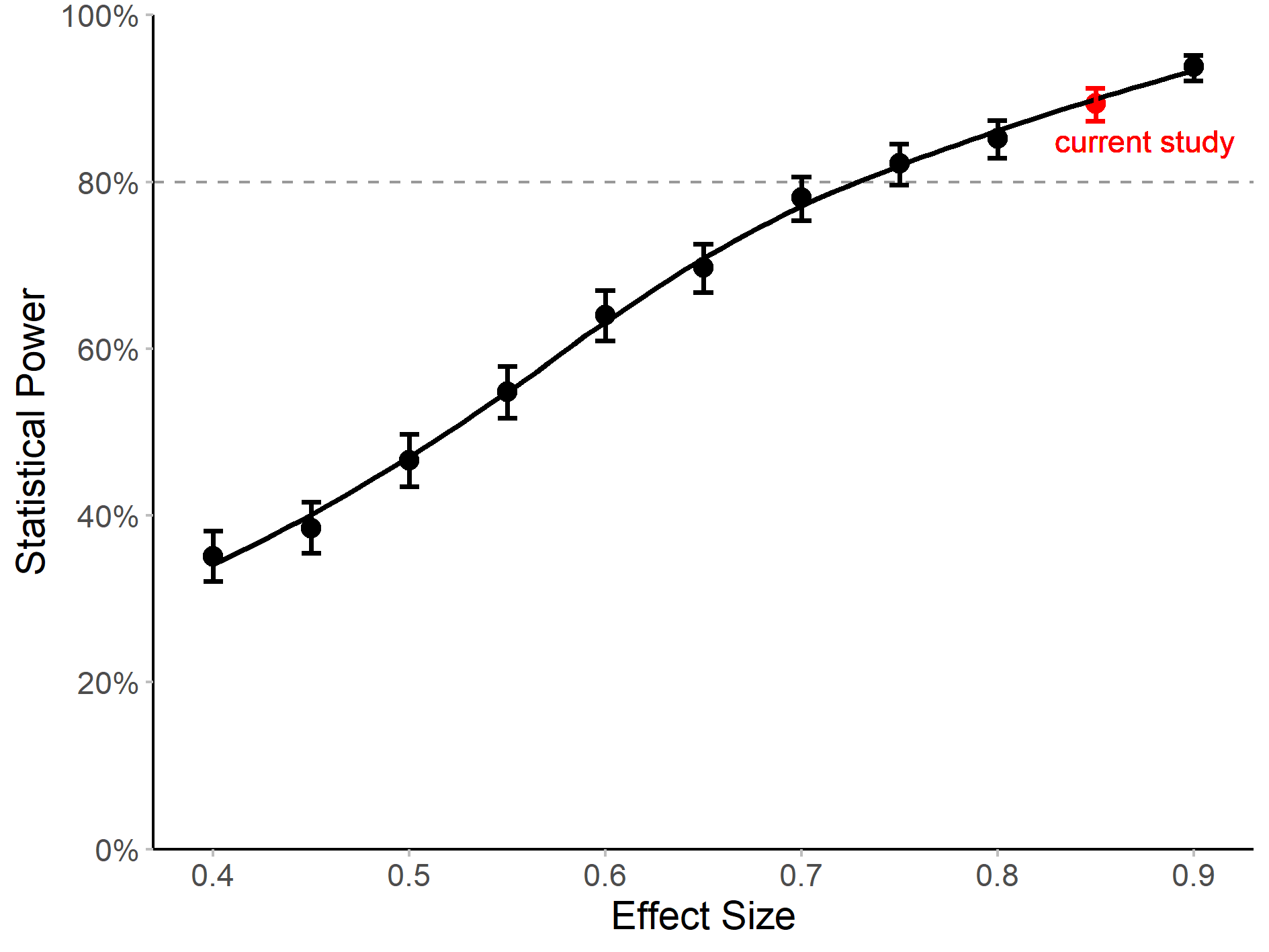


*Figure S1.* Simulation-based sensitivity analysis with statistical power as a function of different effect sizes. The observed effect size and associated power of the current study is marked in red. The dashed line marks an 80%-power threshold. The error bars reflect 95% confidence intervals. Notice that the effect sizes are based on the raw scale of the model and should not be interpreted following rule of thumb guidelines regarding the strength of the effect (e.g., Cohen’s d).

Information about the self-reported questionnaires

Table S1

*Descriptive statistics of the self-reported questionnaires*

| Questionnaire | Mean | SD | Range | Theoretical range | N_missing_ |
| --- | --- | --- | --- | --- | --- |
| RM | 26.05 | 3.77 | 12-34 | 0-37 | 1 |
| LSAS | 34.34 | 20.17 | 0-100 | 0-144 | 10 |
| IRI | 124.10 | 16.03 | 78-165 | 28-196 | 9 |
| PANAS POS | 30.30 | 6.99 | 11-46 | 10-50 | 1 |
| PANAS NEG | 13.44 | 3.42 | 9-25 | 10-50 | 1 |
| DFI | 3.17 | .64 | 1.25-5 | 1-5 | 1 |
| SVO (prosocial) | 66.4% | | | | 9 |

*Note.* RM = Reading the Mind in the Eyes game; LSAS = Liebowitz Social Anxiety Scale; IRI = Interpersonal Reactivity Index; PANAS = Positive and Negative Affect Schedule; DFI = Desire for Future Interaction Scale.

Quantification of physiological synchrony

Two methods that take non-stationarity into account are windowed cross-correlation ^53^ and recurrence quantification analysis ^55^. The latter method is frequently used which has the advantage of having very few assumptions. However, the disadvantage is that it determines synchrony on a binary scale of moments being classified as either synchronized or not. The former method, albeit constrained by more assumptions, has the advantage of differentiating the degree of synchronization by quantifying it on a continuous (correlation) scale. Additionally, we feel that windowed cross-correlation is more intuitive to interpret. Consequently, we decided to apply this method which provides measures of the strength of synchrony and its variability.

The objective of the windowed cross-correlations analysis ^53^ is to calculate the strength of association between two time series while taking into account the non-stationarity of the signals and the lag between responses, that is, to consider the dynamics of a dyadic interaction. Specifically, the time series are segmented into smaller intervals, calculating the cross-correlation for each segment. This allows the means and variances to differ between segments accounting for non-stationarity. This is important as the level of synchrony may change during the experiment, sometimes having moments of strong synchronization while during other times responding less strongly to one another. Additionally, as the strength of association between two time points may differ depending on how far apart they are from each other, the segments are moved along the time series by an increment such that two adjacent segments overlap. Hence, segmenting the time series into smaller intervals and partially overlapping these intervals while moving along the time series provides a better estimate of the local strength of association between the physiological signals of two participants.

Besides the dynamics in the strength of synchronization during the course of the experiment, participants differ in how fast one might respond to a certain event or the other person. In other words, participants might not always be perfectly “in sync” whereby one participant might sometimes respond to the other person or vice versa introducing a delay between the responses of two individuals. To account for this, for each segment, the signals of the two participants are lagged in relation to one another. Specifically, the signal of participant 1 is kept constant while the signal of participant 2 is shifted more and more by a specified lag increment until a maximum lag is reached. Next, the same procedure is performed the other way around with participant 2 being kept constant. The maximum lag determines what is still considered synchrony. For example, if the maximum lag is four seconds, responses from two participants that are four seconds apart from each other are still considered synchronized. On the other hand, if one participant reacts to a certain event and the other participant shows a response 5 seconds later, it is not considered a response to the same event anymore and therefore does not count as synchrony. Based on this approach, there are four parameters that need to be determined: (1) the length of each segment, referred to the window size *w_max_*; (2) the increment with which the segments are moved along the time series, the window increment *w_inc_*; (3) the maximum with which two segments can be lagged from one another, the maximum lag *τ_max_*; and (4) the increment with which two segments are lagged from each other, the lag increment *τ_inc_*. We determined the parameters following an extensive process by comparing previous studies using similar statistical methods, by looking at what is physiologically plausible given the time course of the physiological signals and by employing a data-driven bottom-up approach. Specifically, we first looked into the literature what other researchers have used when calculating physiological synchrony. As the window size is mainly determined by the temporal course of the response, we also looked at studies investigating physiological responses at an individual level. Although there was considerable variation between studies, most studies used response durations between 5 and 10 seconds for both heart rate and skin conductance level ^7,56–58^. Using a different dataset, we performed preliminary analyses with different parameters in this range. As expected, the absolute values of the synchrony measures varied depending on the parameters, but as supported by McAssey and colleagues ^58^, the relative results were not affected (e.g. a dyad manifesting relatively high synchrony showed such tendency for the different parameters). Based on these considerations, we set the parameters as follows: the window size was 8 seconds (160 samples), the window increment was 2 seconds (40 samples), the maximum lag was 4 seconds (80 samples) and the lag increment was 100ms (2 samples).

Calculating the cross-correlations of each lag for each window segment generates a result matrix with each row representing one window segment and each column indicating a lag. The middle column represents the cross-correlation with a lag of zero, while the first and last column contain the cross-correlations for the maximum lag of participant 1 and 2. Hence, the number of columns in the result matrix is (2* *τ_max_* / *τ_inc_*) + 1. The number of rows is given by (*N* − *w_max_* − *τ_max_*)/ *w_inc_*, with *N* being the number of observations in the whole time series.

Based on this result matrix, a so-called peak-picking algorithm is applied. For each segment (i.e., each row in the matrix), the maximum cross-correlation across the lags is detected closest to the zero-lag (i.e., across all columns in a given row). If that maximum correlation is preceded and followed by smaller correlations, it is marked as a peak. For example, if participant 2 synchronizes with participant 1 with a lag of one second, the cross-correlations will become higher the closer the segments from the two participants are shifted towards the point where they are one second apart from each other. When the two signals are lagged by exactly one second the cross-correlation is highest (the peak). If the signals are lagged further away from each other, the cross-correlation decreases again. If, however, a peak cannot be detected, the algorithm assigns a missing value for that segment. This might be the case, for example, if people do not respond to an event or to each other (e.g., both participants wait and do nothing). The peak-picking algorithm outputs a matrix with two columns, containing the value of the maximum cross-correlation (the peak) and the corresponding lag at which the peak cross-correlation is detected. The output has the same number of rows as the result matrix as it searches for a peak cross-correlation for each window segment.

Both the windowed cross-correlations and the peak-picking algorithm are conducted four times per dyad, once for the heart rate responses and once for the skin conductance level responses for the face-to-face condition and for the face-blocked condition resulting in *N_dyads_* * 4 result and peak picking matrices. Finally, the mean of the peak cross-correlations of all window segments (i.e., all rows of the peak picking matrix) is calculated for both physiological measures per Face condition per dyad as the measure of synchrony and is grand-mean centered for the analysis predicting cooperative success.

Please note that the size of the window size is determined by the physiological response rather than by the duration of the trial or the time that participants looked at each other ^53^. The reason for running the analysis per condition instead of per trial was that participants played multiple rounds in a row without intertrial intervals. Therefore, the trials could not be seen as independent events and carry-over effects of synchrony between trials were likely to occur. Additionally, the physiological responses, in particular skin conductance level, are rather slow responses and we expected changes as a result from facing the partner beyond the four seconds (twice the duration of the window size).

Model summary – main analysis

Table S2

*Model summary of the multilevel linear regression analysis predicting cooperation success based on the level of synchrony in heart rate (HR) and skin conductance level (SCL) and their interaction with Face condition (face-blocked = 0; face-to-face = 1). Feedback condition (feedback no = 0; yes = 1) was included as a control variable and Dyad as a random intercept effect.*

|  | **Cooperation success** | | | | |  |
| --- | --- | --- | --- | --- | --- | --- |
| *Predictors* | *Estimates* | *CI* | *t-value* | *df* | *p* | |
| Intercept | 5.07 | 4.85 – 5.29 | 46.65 | 49.31 | **<0.001** | |
| Feedback condition | 0.20 | -0.10 – 0.50 | 1.33 | 48.51 | 0.188 | |
| Face condition | 0.10 | 0.06 – 0.13 | 5.47 | 2890.15 | **<0.001** | |
| HR synchrony | 0.02 | -0.64 – 0.67 | 0.05 | 2668.12 | 0.962 | |
| SCL synchrony | -0.01 | -0.52 – 0.50 | -0.04 | 2884.94 | 0.968 | |
| HR synchrony * Face condition | 0.22 | -0.28 – 0.72 | 0.86 | 2861.92 | 0.389 | |
| SCL synchrony * Face condition | 0.86 | 0.34 – 1.38 | 3.24 | 2882.33 | **0.001** | |
| **Random Effects** | | | | | | |
| σ^2^ | 0.18 | | | | | |
| τ_00_ _Dyad_ | 0.28 | | | | | |
| ICC | 0.61 | | | | | |
| N _Dyad_ | 50 | | | | | |
| Observations | 2905 | | | | | |
| Marginal R^2^ / Conditional R^2^ | 0.033 / 0.619 | | | | | |

*Note.* SCL = Skin Conductance Level; PPN = participant; CI = 95% confidence interval; σ^2^ = residuals; τ_00_ _Dyad_ = random intercept effect for Dyad; ICC = intraclass correlation.

Control analysis – is the level of synchrony an artifact of the experimental set-up?

Because the heart rate and skin conductance level will always show a certain level of synchrony between participants due to the nature of the signals and the experimental set-up ^59^, we conducted a control analysis to show that synchrony was elevated due to the interaction itself. Specifically, we compared the original dyads with newly generated dyads (Player 1 from Dyad*_i_* and Player 2 from Dyad*_i+1_*). Because the trial length varied (there was no time restriction for making a decision), each trial was cut to the shorter trial of the newly generated dyad. Subsequently, the correlation between the responses of the two individuals was calculated per trial per dyad for heart rate and skin conductance level. Finally, we ran an independent t-test on the Fisher-Z-transformed correlation values between the original and the newly generated dyads. As a measure of effect size, we report Cohen’s *d*. The results revealed that for both heart rate and skin conductance level synchrony, the level of synchrony was significantly higher in the original dyads compared to the newly generated dyads (HR: *t*(3622.7) = 8.06, *p* < .001, *d* = .27; SCL: *t*(3015.5) = 4.38, *p* < .001, *d* = .15). This indicates that the level of synchrony was due to the interaction rather than the experimental set-up of the study.

Control analysis – does arousal predict cooperative success?

In the current study we observed that synchrony in skin conductance levels could predict cooperative success. One possible confound is that it is not the synchrony on the dyadic level, but the arousal responses of the two individuals that drive these findings. For example, skin conductance levels might rise if a participant decides to cooperate due to the increased risk of being exploited. Similarly, if the other participant decides to cooperate as well, the same physiological reaction could be expected. Consequently, the responses of the two participants would highly correlate reflecting the individuals’ decisions rather than an interpersonal process. To test this, we conducted a control analysis where cooperation success (the joint points won per trial) was regressed against the participants’ skin conductance level and their two-way interaction with Face condition (face-blocked = 0; face-to-face = 1). For the skin conductance level, we first standardized the responses per participant and then computed the mean skin conductance level per trial. Consistent with the model of the main analysis, we included the Feedback condition (feedback no = 0; yes = 1) as a control variable and Dyad as a random intercept effect. The model summary is displayed in Table S3 which shows that cooperative success could not be predicted by the arousal responses of the two individuals.

Table S3

*Model summary of the control analysis (multilevel linear regression analysis) with participants’ own skin conductance level (SCL PPN) and the interaction with Face condition (face-blocked = 0; face-to-face = 1) predicting cooperation success. Feedback condition (feedback no = 0; yes = 1) was added as a control variable and Dyad was included as a random intercept effect.*

|  | **Cooperation success** | | | | | | | | |
| --- | --- | --- | --- | --- | --- | --- | --- | --- | --- |
| *Predictors* | *Estimates* | *CI* | *t-value* | | | *df* | *p* | | |
| Intercept | 5.15 | 4.97 – 5.34 | | 54.41 | 61.98 | | | **<0.001** |  |
| Feedback condition | 0.12 | -0.14 – 0.38 | | 0.91 | 60.95 | | | 0.362 |  |
| Face condition | 0.10 | 0.07 – 0.13 | | 7.00 | 3566.66 | | | **<0.001** |  |
| SCL PPN1 | 0.00 | -0.02 – 0.03 | | 0.13 | 3591.01 | | | 0.895 |  |
| SCL PPN2 | -0.02 | -0.04 – 0.00 | | -1.64 | 3585.37 | | | 0.100 |  |
| SCL PPN1 * Face condition | 0.02 | -0.02 – 0.06 | | 1.12 | 3603.30 | | | 0.262 |  |
| SCL PPN2 * Face condition | 0.03 | -0.01 – 0.06 | | 1.39 | 3597.28 | | | 0.164 |  |
| **Random Effects** | | | | | | | | | |
| σ^2^ | 0.18 | | | | | | | | |
| τ_00_ _Dyad_ | 0.27 | | | | | | | | |
| ICC | 0.61 | | | | | | | | |
| N _Dyad_ | 63 | | | | | | | | |
| Observations | 3634 | | | | | | | | |
| Marginal R^2^ / Conditional R^2^ | 0.016 / 0.614 | | | | | | | | |

*Note.* SCL = Skin Conductance Level; PPN = participant; CI = 95% confidence interval; σ^2^ = residuals; τ_00_ _Dyad_ = random intercept effect for Dyad; ICC = intraclass correlation.


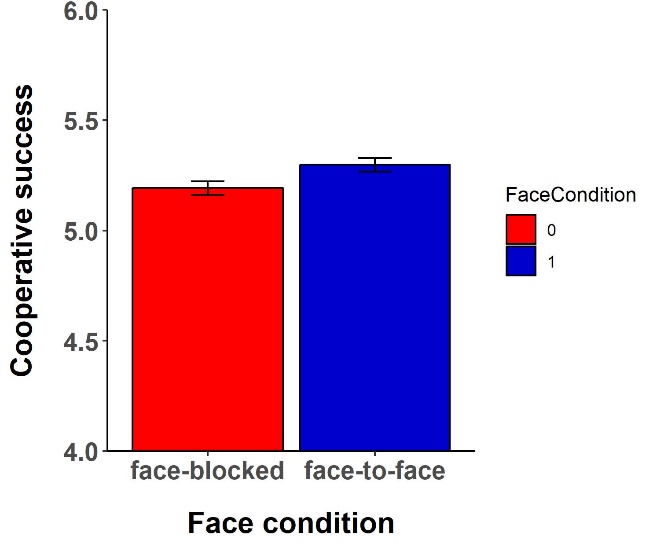


*******

*Figure S2.* The cooperation success rate for the face-blocked and face-to-face conditions with error bars representing 95%-confidence intervals. * *p* < .05; ** *p* < .01; *** *p* < .001.
